# Environmental Awareness for Patients with COPD Undergoing Pulmonary Rehabilitation: Is It of Added Value?

**DOI:** 10.3390/ijerph17217968

**Published:** 2020-10-29

**Authors:** Sara Souto-Miranda, Ana-Carolina Gonçalves, Carla Valente, Célia Freitas, Ana C. A. Sousa, Alda Marques

**Affiliations:** 1 Lab 3R—Respiratory Research and Rehabilitation Laboratory, School of Health Sciences (ESSUA), University of Aveiro, 3810-193 Aveiro, Portugal; sara.souto@ua.pt (S.S.-M.); cfreitas@ua.pt (C.F.); 2 Institute of Biomedicine (iBiMED), University of Aveiro, 3810-193 Aveiro, Portugal; 3 Western Sussex Hospitals NHS Foundation Trust, Worthing PO19 6SE, UK; a.c.goncalves@soton.ac.uk; 4 Pulmonology Department, Centro Hospitalar do Baixo Vouga, E.P.E, 3810-193 Aveiro, Portugal; carlavalente77@hotmail.com; 5 Department of Chemistry, CICECO—Aveiro Institute of Materials, University of Aveiro, 3810-193 Aveiro, Portugal; anasousa@ua.pt

**Keywords:** COPD, pulmonary rehabilitation, education, environment, air pollution

## Abstract

Chronic obstructive pulmonary disease (COPD) is impacted by exposure to environmental contaminants. Improving health literacy on this topic might help to optimize health outcomes. We aimed to design and deliver a health-education session about the impact of environmental contaminants on respiratory symptoms and explore participants’ perceptions on such session. Patients with COPD were recruited from a pulmonary rehabilitation (PR) program. Two focus groups were first conducted to explore knowledge amongst the group. Then, the session was designed and delivered, and three focus groups were conducted to obtain feedback from participants. Data were analyzed thematically by two independent researchers. Thirty-one patients (71 ± 8 years old, FEV_1_ = 47.6 ± 16.8% predicted; 74.2% male) were included. Prior to the session, participants recognized the importance of this topic and described avoidance strategies to deal with symptom triggering due to air pollution. After the session, participants had their knowledge validated, kept some avoidance strategies, but also adapted some “unavoidable” activities of daily living. Patients with COPD value education on this topic, and PR offers a friendly environment to discuss prevention and management strategies. Contents of the session are provided to help deliver these sessions. Future studies could investigate the effectiveness of this intervention on self-management and exacerbations of COPD.

## 1. Introduction

Chronic obstructive pulmonary disease (COPD) is characterized by persistent respiratory symptoms and airflow limitation that is due to airway and/or alveolar abnormalities usually caused by significant exposure to noxious particles or gases [1]. The disease is frequently punctuated by exacerbations, which are episodes of acute worsening of respiratory symptoms that result in additional therapy. Acute exacerbations of COPD are mainly responsible for patients’ clinical deterioration [1,2].

Environmental factors such as exposure to biomass, fossil fuels, metals and indoor and outdoor air pollution, namely sulfur dioxide (SO_2_), nitrogen dioxide (NO_2_) and particle matter (PM), have a key role on the development of COPD and acute exacerbations [3,4,5,6]. The main sources of SO_2_, NO_2_ and PM are the combustion of fossil fuels and aerosols [7]. These pollutants can damage the human airways by increasing bronchial activity, airway oxidative stress and inflammation [8]. Furthermore, PM can also absorb other noxious agents, such as allergens, bacteria and fungi [7]. In a study using the UK Biobank on 303,887 individuals aged 40–69 years, both PM and NO_2,_ have been found to increase the prevalence of COPD [9]. Additionally, exposure to these two pollutants (PM and NO_2_) has been found to increase hospital admissions for acute exacerbations of COPD [8], and general air pollution is perceived by patients themselves as a trigger for COPD exacerbations [10]. Hence, preventive strategies are urgently needed to minimize the contribution of these pollutants for the development and exacerbation of COPD.

Whilst outdoor pollution has been widely studied and has a clear impact on the symptoms of people living with COPD [11], less is known about the impact of indoor air contamination on the daily living of these patients and on the frequency and severity of exacerbations [12]. As exposure to some of these pollutants is a modifiable risk factor, better health literacy around this topic might help people living with COPD to engage in healthier behaviors, and therefore improve their ability to self-manage the condition [13].

Pulmonary rehabilitation (PR) has become a first-choice treatment to manage COPD due to its effectiveness in reducing symptoms and improving physiological, psychosocial and health-related quality of life domains [14,15]. It is a comprehensive intervention, which includes at least both exercise training and education sessions [15]. Typically, these education sessions are related to disease knowledge, management of symptoms and acute exacerbations, PR itself, physical activity, medication, nutrition, sleep and anxiety management [16]. Despite the recognized importance of pollution and air quality on respiratory health, this topic is not usually covered in PR education sessions [16,17]. It is also unknown whether coverage of this topic during education sessions is perceived as needed by patients, and if provided would be of added value. In this new and under-studied empirical context, where there is relatively little prior work developed [18], qualitative research is particularly appropriate, since it represents a unique opportunity to understand people’s beliefs, experiences, attitudes, behaviors and interactions in a controlled environment, before sending a message out to wider audiences [19]. Furthermore, qualitative research has been encouraged, as providing evidence-based healthcare and health policy requires synthesis of knowledge provided by both quantitative and qualitative research [20,21].

The present qualitative study aimed to (1) explore current perceptions of people living with COPD regarding the impact of environmental contaminants on their respiratory symptoms, (2) design an education session to address health literacy needs identified by people living with COPD and (3) explore the perceived benefits and impact of an education session on this topic.

## 2. Materials and Methods

The present study was conducted in a joint collaboration between the Respiratory Research and Rehabilitation Laboratory (Lab3R) of the School of Health Sciences, University of Aveiro (ESSUA), Portugal and the projects RESPIRA and MicroRESPIRA (ref. OHM-E/2015/Proj.6) and PRISMA (ref. OHM-E/2018/1912). This study has been approved by the Ethics Committee of Centro Hospitalar do Baixo Vouga (CHBV; 086092), Portugal. All patients provided informed written consent.

This was a qualitative study, based on the principles of thematic analysis, to explore the perspectives of people with COPD who underwent a PR program. Qualitative studies seek to describe and attribute meaning to a social phenomenon, often through the use of interviews or focus groups [22]. The recognition of these studies as complementary to quantitative studies has been increasing, due to their potential in answering research questions that cannot be answered through quantitative methods and as a vehicle for involving patients in research [22,23,24].

People living with COPD were recruited from the community-based PR program of Lab3R-ESSUA and the primary healthcare center of Estarreja, Portugal. The PR program consisted of supervised exercise training twice per week and education and psychosocial support every other week [13]. Each session lasted approximately 60 min. Education sessions were delivered by a multidisciplinary team (medical doctor, physiotherapy, nurse, nutritionist, psychologist, social worker, environmental toxicologist) and consisted of the commonly discussed topics [11,14,15] with the additional topic of strategies to self-manage symptoms triggered by outdoor and indoor pollution, on a daily basis.

Potential study participants were included if they had a diagnosis of COPD confirmed by spirometry (forced expiratory volume in one second/forced vital capacity <70) and were in a stable state of the disease (no acute exacerbations in the previous 4 weeks) [16]. Using a pragmatic approach, a convenience sample [17] of two focus groups (with 6 to 7 participants each) was determined to explore current perceptions of people living with COPD on the impact of indoor and outdoor air pollution, and inform the design of the education session on air quality and respiratory symptoms. The education session was then designed and delivered in a group session by an expert environmental toxicologist and experienced respiratory physiotherapist. A total of three focus groups (6 participants each) were then conducted, aiming to explore the perceived value of the education session, added to the PR program, and identify any changes that might have been adopted by participants after the education session. All focus groups were conducted by the respiratory physiotherapist and followed a semi-structured topic guide. No more focus groups (before or after the education session) were necessary as data saturation was reached, that is, the same ideas and discourse content of the participants were becoming repetitive, and therefore additional data collection was not considered necessary at this stage [25].

Sociodemographic and clinical data were collected to characterize the sample before each focus group.

Audio recordings of the focus groups were transcribed verbatim. Transcriptions were conducted by one researcher and checked for accuracy by a second researcher using the audio recordings. Thematic analysis was conducted, as recommended by Braun and Clarke, by two independent researchers and consisted of identifying, analyzing and reporting patterns/themes found in data [24]. Reflection memos were used throughout the analysis to assure credibility. The final themes were agreed by the two researchers who were familiar with the data set.

Descriptive statistics were computed with SPSS Statistics software version 25 (IBM, SPSS inc., Chicago, Illinois), for the purpose of sample characterization. Nvivo software [19] was used for qualitative data management.

## 3. Results

### 3.1. Sample Characteristics

Thirty-one people living with COPD participated in the study, that is, 13 in the two initial focus groups and 18 in the three focus groups after the education session. The mean duration of the focus groups was 45 min. Most participants were male (*n* = 23; 74.2%), with an average age of 71 ± 8 years old, GOLD 2 (*n* = 13; 41.9%) and 3 (*n* = 14; 45.2%) according to airflow limitation (FEV1 = 47.6 ± 16.8% predicted) and GOLD B (*n* = 18; 58.1%) according to assessment of symptoms and exacerbation risk. Table 1 shows the clinical characteristics of the participants.

### 3.2. Focus Groups before the Education Session on Indoor and Outdoor Air Pollution

Three themes can explain findings from these two focus groups.

#### 3.2.1. Recognizing Air Quality as an Unfortunate Regional Problem

Participants described the region where they lived as a place where air quality was poor, due to the wet coastal weather and the presence of heavy industry and agriculture. Participants speculated that other areas of the country, with less industrial activity, would not be as affected, and that there was little they could do to change it. Most felt it should be the responsibility of local authorities to prioritize safety measures for the local businesses and industry in order to improve the quality of air locally. Participants described “every-day evidence” for the poor quality of the air in their region and appeared to stay unanimous as a group when describing air quality as a regional problem.

*“Look, I went once to a respiratory treatment center, and do you know what I was told there? That the majority of patients were from our region!”*

(P3, M, 69 years old).

#### 3.2.2. Poor Air Quality as a Cause of Respiratory Symptoms

Even before the education session on the effects of environmental contaminants, all participants were aware of environmental causes for having worse respiratory symptoms. Participants often felt anxious about the possibility of exacerbating the disease by being exposed to pollutants. These included smoke from factories; crowded places (particularly where people smoked); strong odors, both indoors and outdoors; smoke from locals burning wood or farming activities; dust from local roads; or from using fireplaces at homes as their form of heating during the winter.

*“Every time the neighbors make a fire in their backyard to burn wood, or tires… I don’t know what they burn there, but I have to lock myself in the house (…) I feel anxious and breath heavily, I feel that smoke is bad for me”*

(P1, F, 76 years old).

Everyday chores such as housekeeping were also identified as symptom triggers, as they involved contact with dust and strong odors from cleaning products.

*“At home, when my wife (…) uses sprays I have to run away. Actually, she knows, everything must be natural for me. When she vacuums or cleans the house, I have to escape”*

(P2, M, 78 years old).

#### 3.2.3. Avoidance as the Only Strategy

Before the education session, participants gave various examples on how they avoided contexts of poor air quality, which trigger their respiratory symptoms. Some strategies were simple/short-term and were already noted in the previous theme (locking themselves in the house or walking out if needed). Other strategies of avoidance were more long-term, such as moving home to a region perceived as less polluted, or with better weather.

*“I was forced to move out of my region, and move [house, elsewhere]”*

*Researcher: “Why did you move?”*

*“[Because] I cannot be in places with a lot of humidity, particularly in the winter, it was just horrible”*

(P2, M, 78 years old).

### 3.3. Contents of the Education Session on Air Quality

The themes above highlighted participant concerns about the air quality and that they had already identified many environmental-related triggers to their symptoms, both indoors and outdoors, reinforcing the relevance and need for an education session. When asked about strategies they were currently using to overcome their symptoms, participants had focused on avoiding harmful triggers, and there were no suggestions on how to adapt routine activities of daily living to minimize the impact of air quality on respiratory symptoms. Based on those findings, an education session was tailored to the group. The session focused on a variety of possible strategies (reinforcing avoidance, but also suggesting simple adaptations that can be made to activities of daily living), aiming at assisting participants to self-manage their respiratory symptoms, in the presence of environments with poor air quality. Some of the strategies discussed during the education session are presented in Table 2. Additionally, a leaflet was given to patients with indoor recommendations (see Supplementary Materials). The session lasted 60 min.

### 3.4. Focus Groups after the Education Session

Overall, participants found the education session on this topic to be beneficial. The session was considered a good learning opportunity. While some participants continued to use only avoidance strategies they had already adopted prior to the session, others explained how they adapted simple activities of daily living to minimize triggers to their symptoms. The two themes below explain in more detail the findings of the focus groups completed after the education session.

#### 3.4.1. Validating Previous Knowledge and Experience, and Learning Something New

Although participants had good knowledge prior to the session of the impact of environmental contaminants on their breathing and were able to identify triggers to their own symptoms, they appeared to have enjoyed the validation that the session provided to their knowledge on this topic.

Participants expressed they found the session a good opportunity to learn something new, became more alert for the importance of environmental contaminants, and some revealed how they had shared knowledge with others.

*“This session was an eye opener. Because there are things I never thought to be harmful”*

(P9, M, 65 years old).

*“We learnt a lot and I am now transmitting it to others. I have already lent those papers [leaflets] to others, so that they can photocopy them”*

(P2, M, 78 years old).

#### 3.4.2. Keeping Avoidance Strategies and Adapting Tasks and Chores that Cannot Be Avoided

After the education session, there were several participants who did not make any changes to their daily routine as a result of what they had learnt in the session. Some felt it was difficult to change their routine; others believed the avoidance strategies they were using before the session were enough, and they did not feel the need of making further changes. Some also expressed the increased costs of some of the adaptations as a barrier to change.

Other participants, however, focused on some of the advice given during the session and explained they had made changes to the tasks they could not, or did not wish to, avoid.

*“I now wear a mask to do the pigeon keeping. I had to adapt [try a few different masks] because some masks did not work for me. But now I use one and then throw it away straight after”*

(P2, M, 78 years old).

Some of those changes were also adopted by their family members living in the same household, a result from the knowledge participants shared after the education session, demonstrating the wider impact these sessions may have on patients and families.

*“My relatives are now a little more alert and more cautious about using sprays [in the house]”*

(P20, M, 73 years old).

## 4. Discussion

To the authors’ best knowledge, this was the first study to explore the views of people living with COPD regarding the impact of indoor and outdoor contaminants on their respiratory symptoms and on their daily life. It also provides guidance to healthcare professionals running PR programs on the potential use of an additional education session on this topic, of increased interest to the public. The present study has shown that people living with COPD value education sessions on environmental contaminants as a way to validate their knowledge on the topic, but also to add on their available strategies to prevent symptom exacerbation and better manage activities of daily living.

Although scarce evidence exists on the impact of environmental education on health outcomes of people living with COPD, studies including people living with other respiratory conditions, such as asthma, report similar findings to those described in the present study. People living with asthma and their loved ones have highlighted the need for education sessions on this topic [26], and a possible decrease in the use of health resources has been suggested after environmental education in this population [27]. Moreover, since environmental contaminants may be a cause of exacerbations, this type of preventable action aiming to increase patients’ knowledge to target modifiable risk factors has also been advocated for people with lung cancer [28] and people with idiopathic pulmonary fibrosis [29]. However, the effectiveness of such interventions in these diseases and COPD requires further investigation.

Since air pollution and its impacts on health are recognized by international societies, such as the Forum of International Respiratory Societies and the World Health Organization, new studies exploring prevention and control strategies for the defense of respiratory health have been recommended [30,31]. It is likely that the increased public awareness, including of those living with respiratory conditions, will lead to a higher number of people having an interest on this topic. Thus, PR offers a friendly environment to address this topic, develop prevention/management strategies and establish the effectiveness of this intervention.

It is of note that, although participants valued the session delivered on air pollution, many did not change behaviors and chose to keep avoidance as the only strategy, suggesting health education might not be enough to produce behavior change in patients with COPD. Fear and avoidance of triggers have been recognized as poor strategies to deal with diseases [32,33], and their recognition without the necessary skills to mitigate them can lead to a reduced quality of life [34]. Hence, add-on interventions to education on this topic, such as individual counseling and self-management programs, might be necessary to assist behavior change [35]. To the authors’ best knowledge, there is currently no evidence on programs developed for triggers education and coping strategies to deal with pollutants. Therefore, future research is needed.

Although environmental education might play an important role in improving health literacy and self-management, policy and patient advocacy interventions are needed [30,36]. More severe policies, such as restricting the combustion of fossil fuels and the integration of harsh chemicals in house products, might need to be placed to ensure the safety and well-being of respiratory patients.

The present study was not conducted without limitations. Participants were recruited from one single geographical area and often focused on specific problems of their region. It is possible that people living with COPD in other locations might have described the impact of air pollutants on their daily lives differently. Therefore, additional qualitative research in other parts of the world could be enlightening. The contents of the education session here presented were selected based on the needs identified by participants before the session. While professionals delivering PR in other parts of the world are welcome to use the content here presented to deliver their own education sessions on environmental contaminants, it is recommended that feedback from participants is sought, so that sessions can be adequately tailored to the needs of those attending. Lastly, future studies may consider exploring the impact of this intervention on self-management and other health-related outcomes (i.e., quality of life and frequency or severity of acute exacerbations of COPD).

## 5. Conclusions

People living with COPD value education about the impact of air pollution on their respiratory symptoms, and PR is an appropriate setting to deliver on this high-interest topic. People living with COPD tend to use avoidance strategies to prevent symptoms triggered by air contaminants. Education sessions on strategies to adapt activities of daily living that cannot be avoided are acknowledged as needed and well received by those attending PR.

## Figures and Tables

**Table 1:** Characteristics of participants with chronic obstructive pulmonary disease (*n* = 31).

	Patients (*n* = 31)
Age, years (mean ± SD)	71.4 ± 7.5
Gender, *n* (%)	
Female	8 (25.8)
Male	23 (74.2)
Years of formal education, *n* (%)	
None	1 (3.2)
1 to 4	17 (54.8)
5 to 9	5 (16.1)
10 to 12	4 (12.9)
≥13	4 (12.9)
GOLD (1–4), *n* (%)	
1	3 (9.7)
2	13 (41.9)
3	14 (45.2)
4	1 (3.2)
GOLD (A–D), *n* (%)	
A	5 (16.1)
B	18 (58.1)
C	0 (0)
D	8 (25.8)
Charlson Comorbidity index (mean ± SD)	4.2 ± 1.1

GOLD: global initiative for chronic obstructive lung disease; 1–4 airflow limitation: 1—FEV1 ≥ 80, 2—FEV1 50–79, 3—FEV1 30–49, 4—FEV1 < 30; A–D (ABCD) assessment tool: assessment of symptoms and exacerbation risk: A—0–1 exacerbations without hospital admission and COPD Assessment Test (CAT) < 10, B—0–1 exacerbations without hospital admission and CAT > 10, C—> 1 exacerbation, or 1 exacerbation leading to hospital admission and CAT < 10, D— > 1 exacerbation, or 1 exacerbation leading to hospital admission and CAT > 10.

**Table 2:** Description of the education session on the environment, delivered in the community-based pulmonary rehabilitation program to people living with chronic obstructive pulmonary disease.

Aspect to Address	Practical Strategies Suggested and Discussed	
**Outdoors**		
Cigarette smoke	Avoid places where smoking is permitted.	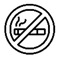
Places with known high pollution levels	Use of face masks.	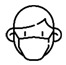
Car pollution	When possible, choose less busy roads when driving or walking.	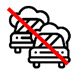
High pollution levels	Check your local air quality predictions when planning outdoor activities.	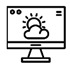
Outdoor exercise	If you exercise outdoors, avoid hours where the traffic is high.	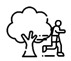
**Indoors**		
House dust	Use a vacuum cleaner or a wet cloth instead of a broom or duster	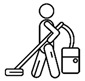
	Avoid using carpets and curtains.	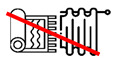
Chemicals in activities of daily living (e.g., bleach, mixing different cleaning products, air fresheners and antibacterial products)	Use soap, lemon juice or vinegar instead.	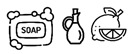
Odors and chemicals brought into the house	Change your clothes and shoes before entering home.	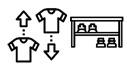
Activities that produce smoke	Avoid tobacco smoke, open fireplaces including their cleaning, use of pesticides, burning candles or incense.	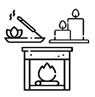
House ventilation	Open windows every day with the exception of when there is smoke from fires, high levels of pollens, alerts of bad air quality by the authorities, and if living near a busy road.	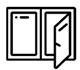

Icons were downloaded free of charge from www.flaticon.com.

## Supplementary Materials

The following are available online at https://www.mdpi.com/1660-4601/17/21/7968/s1, S1: Educational leaflet—Importance of the environment on respiratory diseases.
