# Supplementary material for: Environmental Awareness for Patients with COPD Undergoing Pulmonary Rehabilitation: Is It of Added Value?
Source: Int J Environ Res Public Health. 2020 Oct 29;17(21):7968. doi: 10.3390/ijerph17217968 (PMC7663233; doi:10.3390/ijerph17217968)
Supplement: Supplementary file 1 [file ijerph-17-07968-s001.pdf]

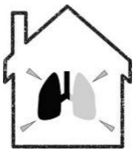

Importance of the environment on respiratory diseases

The importance of the indoor environment

We stay most of our time inside buildings, especially in our house. That is why air quality in our houses should be the best possible.

Some of the activities we do in our homes can have a negative impact in indoor air quality and consequently, our health.

In this leaflet, we are providing you with information on how to improve the air quality of your home.

House dust

We know that house dust contains several products. Some of them can be toxic and can affect your respiratory health. That is why you should avoid being exposed to dust.

How can I do it?

1. Vacuum the house

Sweeping the dust does not eliminate the dust from your house. It only moves the dust from one place to another. Thus, avoid sweeping and choose to vacuum every time you can.

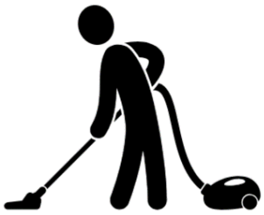

The vacuum cleaner should have a filter so the dust gets trapped. Almost all modern vacuum cleaners come with a filter.

What if I don’t have a vacuum cleaner?

You should use a damped cloth around the broom or a wet mop instead.

2. Cleaning the dust

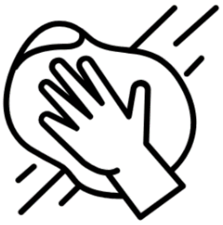

Wipe the surfaces/furniture with a damped cotton-based cloth. You do not need to use any cleaning product.

3. Avoiding carpets/rugs

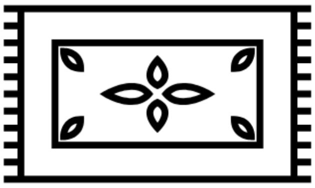

Carpets and rugs concentrate the dust and all its contaminants. If possible, do not use any carpets/rugs. If you have no choice, opt for those that can be regularly washed.

4. Curtains

Similar to carpets, curtains also accumulate dust. Try to clean them frequently.

Cleaning products

Several cleaning products are irritant to the airways (for example bleach). Others have ingredients that might be toxic and therefore should not be used.

How can I do it?

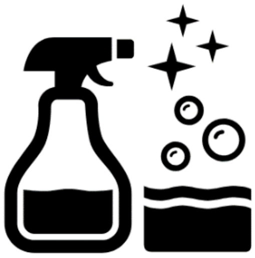

1. Do not use bleach. If you really need to use it, then ventilate well the area where you used it.

2. Never mix cleaning products. When mixed, some cleaning products originate dangerous compounds for your health.

3. Do not use air fresheners. These products contain substances that might be toxic.

4. Before using a cleaning product, think if you really have to use it, or if you can choose a more natural option. **Traditional soap, lemon and vinegar** are good alternatives.

5. Do not use antibacterial detergents or soaps. In one hand by using them you are only enabling the resistant bacteria to survive, and on the other hand these products have a substance that interferes with your hormones, which might have a negative impact on your health.

Personal care products/ cosmetics

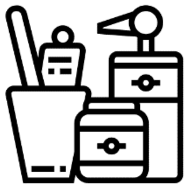

Some cosmetics and hygiene products contain ingredients that might be irritant or toxic, and therefore should be avoided.

How can I do it?

1. Do not use spray products. Generally, there are alternatives for spray products, such as deodorants and hair lacquers. However, if you really need to use spray products, be sure to ventilate well the room.

2. Avoid products that have intense scents, such as perfumes. Some moisturizers and lotions also have intense scents and should be avoided.

Avoid activities that release toxics

Some day-to-day activities might release toxic products inside your house. Try to avoid those activities.

1. Do not smoke inside the house and **do not allow** others to smoke inside your house. Exposure to tobacco even when smoked by others has negative effects on your health.

2. Fireplaces are an important source of contaminants. If you use your fireplace to cook or as a source of heating and are not able to stop doing it, make sure your chimney is well cleaned and that it has good air extraction. You should also be careful and use only natural wood and no other products such as plastics, rubber or wood with paints or varnishes.

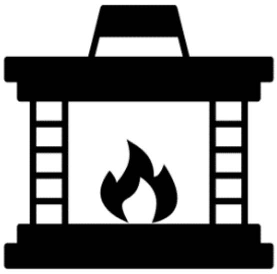

3. Never try to clean the chimney of the fireplace. Call a professional to do it. In case you can't afford to call a professional, please ask a person without respiratory disease and who knows how to do it with personal protective equipment (mask and goggles are mandatory).

4. Do not use pesticides in interior plants, nor to kill ants or flies. These products are highly toxic and remain in the house for a long period after use.

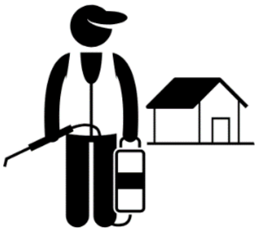

5. Do not burn candles or incense inside the house. If you really have to do it, make sure to open the windows so the toxic products do not remain in the house.

Avoid bringing toxic products inside your home

A lot of the toxics that we have inside our houses are brought by us from outdoors. Avoiding their entrance in our house is important.

How can I do it?

1. If you were on your backyard applying pesticides, **you should not enter your house with the same clothes or shoes.**

2. If you work with dangerous chemicals, you should be cautious **and change your clothes and shoes before entering your house.**

3. If you are already used to not using the same shoes at home, try to change your **shoes at the door** instead of changing them only when you go to your room. By doing this, you are not spreading the chemicals you bring in your shoes. If you are not used to this, try to implement this routine.

Air the house

Contrary to our believes, sometimes our indoor environment is more contaminated than the outside. Thus, with some exceptions**, it is important to ventilate your house daily. Ventilating the house is particularly important in humid houses, to avoid the appearance of fungi.

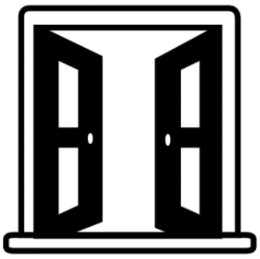

How can I do it?

1. Open the windows of your bedroom when you wake up.

2. Never cover the kitchen ventilator.

3. After showering and every time possible air the bathroom.

4. If you have a tumble dryer, try to place it in a room that you can ventilate frequently.

**Situations when you should not air the house

There are some situations when the outdoor environment is very contaminated, and you should not open the windows of the house.

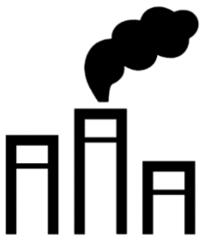

1. When the authorities alert that there will be bad air quality

Sometimes, outdoor air quality is poor due to high levels of particles and ozone. In these cases, the local authorities usually alert the

population though the media, television, radio, etc. During that period, you should avoid opening windows and drying your clothes outside.

2. When there is a high concentration of pollens in the air.

Sometimes during the year, such as in Spring, there is high concentration of pollens in the air. During those periods you should also avoid opening windows and drying your clothes outside.

3. During wildfires

You should also avoid opening windows when there is a lot of smoke in the air due to the wildfires. If the fire is near your residence, then you should follow the advice of the local authorities.

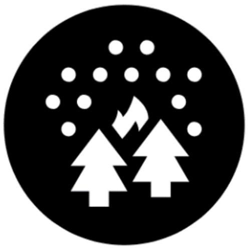

4. If your house is near a highway or a busy road.

Traffic releases smoke that is toxic for your respiratory health. You will have to find a balance. Try to air your house when there is less traffic instead of leaving the windows opened for a long period of time.
